# A residential labeled dataset for smart meter data analytics

**DOI:** 10.1038/s41597-022-01252-2

**Published:** 2022-03-31

**Authors:** Lucas Pereira, Donovan Costa, Miguel Ribeiro

**Affiliations:** 1 ITI, LARSyS, Técnico Lisboa, Lisbon, 1049-001 Portugal; 2 University of Madeira, Faculty of Exact Sciences and Engineering, Funchal, 9020-105 Portugal

**Keywords:** Electrical and electronic engineering, Energy grids and networks

## Abstract

Smart meter data is a cornerstone for the realization of next-generation electrical power grids by enabling the creation of novel energy data-based services like providing recommendations on how to save energy or predictive maintenance of electric appliances. Most of these services are developed on top of advanced machine-learning algorithms, which rely heavily on datasets for training, testing, and validation purposes. A limitation of most existing datasets, however, is the scarcity of labels. The SustDataED2 dataset described in this paper contains 96 days of aggregated and individual appliance consumption from one household in Portugal. The current and voltage waveforms were sampled at 12.8 kHz, and the individual consumption of 18 appliances was sampled at 0.5 Hz. The dataset also contains the timestamps of the ON-OFF transitions of the monitored appliances for the entire deployment duration, providing the necessary ground truth for the evaluation of machine learning problems, particularly Non-Intrusive Load Monitoring. The data is accessible in easy-to-use audio and comma-separated formats.

## Abstract (summary)

**Table:** 

Measurement(s)	Electricity Consumption
Technology Type(s)	Transformer Device
Sample Characteristic - Location	Portugal

## Background & Summary

Smart-meter data analytics has gained traction in the past years, leveraged by the massive deployments of smart meters worldwide. For instance, in^1^ it is stated that in the United States, electric utilities aimed at installing around 90 million smart meters by 2020. Also, it was expected that almost 72% of European consumers would have a smart meter in the European Union, which would represent a roll-out of close to 200 million smart meters.

The type of problems to tackle and the employed data analytics methods are extensive when it comes to smart meter data, as highlighted by some literature reviews on the topic, e.g.,^2–5^. In the concrete case of the residential sector, smart-meter data applications include real-time and historical feedback^6^, forecasting^7^, appliance and activity recognition^8,9^, anomaly detection^10^, and demand-side flexibility estimation^11^. In this context, electricity consumption datasets are crucial to test the signal processing and machine learning algorithms at the core of such applications.

Several residential electricity consumption datasets can be found in the literature, each of which with its own characteristics as summarized in different survey papers^12–14^. Such characteristics include the number of sensors (e.g., a single sensor for the whole building, circuit-level, and appliance level), type of measurements available (e.g., current, voltage, active and reactive power, and energy tariffs), data granularity (e.g., from several kHz to one sample every hour or less), and dataset duration (e.g., from a couple of days to several years)^15^. While all these characteristics play an important role in classifying the different datasets, the co-existence of aggregated and individual appliance consumption measurements is commonly used to categorize electricity consumption datasets since this aspect has a crucial implication on the potential applications of each dataset^13^. For example, algorithms for appliance identification and activity recognition can only be evaluated in datasets where individual appliance consumption data are also available. Fortunately, this is the case with the majority of the existing residential datasets, as over 20 of them include both types of measurements, e.g., the Reference Energy Disaggregation Dataset (REDD)^16^, Almanac of Minutely Power dataset (AMPds)^17^, REFIT^18^, and UK-DALE^19^.

Besides the monitored electrical quantities, for some application areas, the existence of labeled appliance transitions (also referred to as power events) is essential to train and validate the underlying algorithms. This is the case of real-time appliance recognition algorithms that rely on the accurate detection and classification of appliance transitions^20,21^, and anomaly detection algorithms that often rely on historical patterns of appliance transitions^22,23^. Still, to the best of our knowledge, to date, there are only four real-world datasets that contain labeled appliance transitions, namely, Building-Level Fully-labeled dataset for Electricity Disaggregation (BLUED)^24^, SustDataED^25^, Energy Monitoring through Building Electricity Disaggregation (EMBED)^26^, and Fully-labeled High-Frequency Electricity Disaggregation Dataset (FIRED)^27^.

The BLUED dataset consists of voltage and current measurements for a single-family residence in the United States. BLUED contains seven consecutive days of data, sampled at 12 kHz. Every state transition of each appliance in the home was labelled and time-stamped. SustDataED consists of electric energy consumption and room occupancy measurements taken from a single-family apartment in Portugal during ten consecutive days. The voltage and current measurements were sampled at 12.8 kHz. The dataset also contains the individual consumption for 17 individual loads, measured at 0.5 Hz complemented with individual labels for the state transitions of those loads. The EMBED dataset contains the aggregate power measurements and load data of different appliances for three residential units in the United States. The data was collected for at least two weeks in each household. The voltage and current measurements were sampled at 12 kHz, whereas the individual load measurements were sampled at 1–2 Hz. The FIRED dataset contains 52 days of 8 kHz aggregated current and voltage measurements of a 3-phase residential apartment in Germany. The dataset also contains the individual appliance measurements of 21 appliances, sampled at 2 kHz, with labelled power consumption transitions. Finally, it should be stressed that there are a few other labeled datasets, however, these were obtained either in controlled environments Plug-Load Appliance Identification Dataset (PLAID)^28^, Laboratory-measured IndustriaL Appliance Characteristics (LILAC)^29^ and LIT^30^), or through simulation (Synthetic Energy Dataset (SynD)^31^ and LIT).

Against this background, this paper introduces a new real-world labelled dataset, the SustDataED2. The SustDataED2 is the second iteration of the SustDataED dataset and was collected on a second household for a longer period. More precisely, SustDataED2 contains 96 days (from October 6th 2016 to January 9th 2017) of aggregated and individual appliance consumption from one house with three residents. The current and voltage waveforms were sampled at 12.8 kHz, and the individual consumption of 18 appliances was sampled at 0.5 Hz. The dataset also comprises power measurements derived from the current and voltage waveforms, namely, active power, reactive power, current, and voltage. These measurements are made available at 50 Hz and 1 Hz.

This paper provides a thorough description of how the dataset was collected and labelled. It includes detailed information on how the collected data was pre-processed from the original files and organized to form the SustDataED2 dataset. This paper also analyzes the quality of the data and provides instructions on how to reuse the dataset.

## Methods

### Data collection setup: aggregated consumption

The setup for collecting aggregated consumption consists of a multi-channel data acquisition board (LabJack U6 [see http://www.labjack.com/U6, accessed 13/09/2021]), one processing unit (Toshiba NB300 [see https://www.pcworld.idg.com.au/review/toshiba/nb300/338720/, accessed 13/09/2021]), and a combination of split-core Current Transformers (CTs)) and Voltage Transformers (VTs). The selected CTs were of the model SCT-013-050 (see http://www.datasheet-pdf.com/PDF/SCT-013-050-Datasheet-YHDC-1328320, accessed 13/09/2021) with a 50 A to 1 V voltage output, to ensure direct compatibility with the DAQ. These were not only the cheapest CTs on the market but also the less intrusive due to the fact they have a split-core which makes the installation easier. As for the VT, at the time of development, there were no feasible alternatives on the market. Therefore it was necessary to develop a custom solution. In this concrete case, the developed transformer steps down the voltage from 230 V to 0.5 V RMS, ensuring full compatibility with the data acquisition device. The LabJack U6 was selected because, at the time of development, it offered the best trade-off between functionality and price. In particular, the fact that it supported a sampling rate up to 50 Hz with 16-bits resolution was vital since it allowed the collection of current and voltage waveforms at high frequency. Furthermore, since LabJack support USB-3, it could be directly connected to any computer to handle all the computation tasks. In this case, the Toshiba NB300 notebook was selected since it was already available from a previous project.

**Fig. 1:**
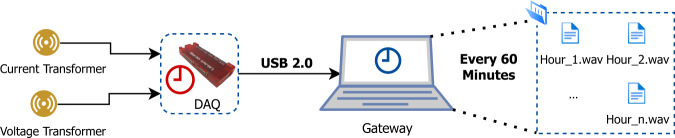
Main components of the data collection setup for the aggregated consumption (icons by draw.io).

Figure 1 illustrates the main components of the aggregated consumption data collection platform. The CT and VT are installed in the main breaker box, hence measuring the total household consumption. The DAQ performs the data acquisition at a pre-defined sampling rate (12.8 kHz in this case) and sends the samples to the gateway via USB 2.0. The sampled current and voltage waveforms are stored in the Energy Monitoring and Disaggregation Data Format (EMD-DF) file format^32^ in one-hour long files. This was done to mitigate the effects of synchronization issues that may occur due to the differences in the internal clocks of the data acquisition (LabJack U6) and processing unit (Toshiba NB300) devices (see https://goo.gl/GTMp9Y, accessed 20/01/2022). Ultimately, instructing the data acquisition software (running on the processing unit) to store the collected samples every hour on a new file ensures that any synchronization issues are not propagated through time.

### Data collection setup: appliances consumption

The appliance-level data collection was performed using the Plugwise system (see https://www.plugwise.com/, accessed 13/09/2021), which was also used in^25,33,34^.

**Fig. 2:**
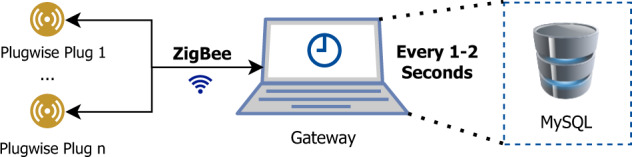
Main components of the data collection setup for individual appliance consumption (icons by draw.io).

Figure 2 illustrates the main components of the individual data collection platform for individual appliances. The Plugwise sensors are connected between the appliances to be monitored and the respective power outlets. The gateway (Toshiba NB300) requests the latest power measurement in each of the plugs through the ZigBee (see http://www.zigbee.org, accessed 13/09/2021) protocol, using the python-plugwise library (see https://pypi.org/project/python-plugwise/, accessed 13/09/2021). The collected samples are stored in a local relational database. It should be noted that the plugwise sensors report their consumption sequentially, meaning that the first plug is only revisited once all the remaining plugs have been visited. Each plug visit takes around 100 ms, meaning that it takes one second to scan ten plugs (1 Hz) when all the plugs are online. In the case of SustDataED2, since there are 18 plugs, each appliance will be scanned roughly every two seconds (0.5 Hz). Ultimately, this also means that the timestamps collected for each will not necessarily be the same. For example, if the scan starts exactly at 12:00:00, the first ten plugs to be visited will have a timestamp of 12:00:00, whereas the remaining eight will have a timestamp of 12:00:01.

### Data labelling

In order to label the individual appliance transitions, we relied on the semi-automatic labelling platform described in^35^. More precisely, event detection algorithms are executed in the background to locate each appliance’s power events. The events are then presented to the end-user in a graphical user interface for correction, i.e., remove false positives and false negatives. In the case of SustDataED2, the first author was the person responsible for visually inspecting the system detected labels for validation and correcting any erroneous detections (i.e., false positives and false negatives). Finally, the only labelling criteria was that any power event with an absolute power change of at least 10% of the appliance consumption mode (excluding zeros) was considered for labelling. The amount of power change was calculated by subtracting the average power before and after each potential power event, t. E.g., if the sample just before the event of interest is 20 Watts, and the one just after the event is 50 Watts, the calculated power change is 30 Watts.

### Deployments

The monitoring platform was deployed in a single-family house (three adults) for three months (between October 6th 2017 and January 9th 2017).

The monitored house, built in the 1910s, comprises nine main divisions across two floors. Eighteen appliances were monitored across six divisions (two bedrooms, office, kitchen, living room, dining room, and one WC). It was impossible to monitor the appliances in the remaining three divisions due to the limited coverage range of the ZigBee protocol. Table 1 lists the monitored appliances and the respective monitoring periods.

**Table 1:** List of monitored appliances and the respective monitoring periods.

ID	Appliance	Start Date	End Date
1	Coffee Machine	2016-10-06	2017-01-09
2	Fridge - Freezer	2016-10-06	2017-01-09
3	Freezer	2016-10-06	2017-01-09
4	Hand Mixer	2016-10-06	2016-12-13
5	Hair Dryer + Straightener	2016-10-06	2017-01-09
5	Kettle	2016-10-06	2017-01-09
7	MacBook 2007	2016-10-06	2017-01-09
8	MacBook Pro 2011 (1)	2016-10-06	2016-11-30
9	MacBook Pro 2011 (2)	2016-10-06	2016-11-25
10	Microwave	2016-10-06	2016-12-09
11	Stove + Oven	2016-10-06	2017-01-09
12	TV Philips	2016-10-23	2016-11-26
13	TV Sharp	2016-10-06	2016-10-30
14	TV Grundig	2016-10-06	2016-10-23
15	TV Samsung	2016-11-26	2017-01-09
16	TV-LG	2016-10-06	2017-01-09
17	Toaster	2016-10-06	2016-12-09
18	Vacuum Cleaner	2016-10-06	2017-01-09

## Data Records

**Fig. 3:**
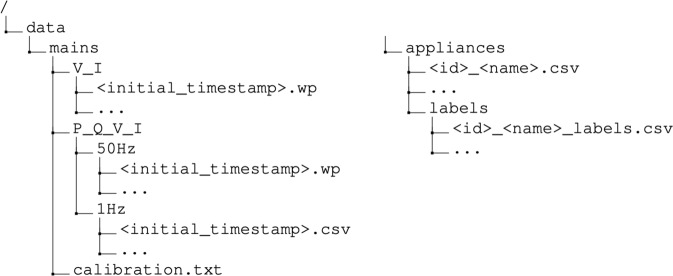
Underlying folder and file organization of SustDataED2 Dataset.

The SustDataED2 dataset is made available in the form of Sony Wave64 (W64) and Comma Separated Values (CSV) files. The data is available on the Open Science Framework (OSF) data repository at 10.17605/OSF.IO/JCN2Q^36^. Figure 3 shows an overview of the underlying organization of SustDataED2. The following subsections describe the contents of the different files.

### Aggregated consumption measurements

Aggregated consumption data is made available in two different ways: 1) raw (voltage and current), and 2) processed (active power, reactive power, voltage RMS, and current RMS).

#### Raw data

The raw data files are available under the folder “mains/V_I”. The voltage and current waveforms are stored in the W64, with a sampling rate of 12.8 kHz. In order to reduce the file size, the W64 files were compressed using the WavePack (see https://www.wavpack.com/, accessed 13/09/2021) audio compression library (extension *.wp). For details on the decompression procedure, please refer to the Usage Notes section for more details.

The name of each file consists of a Unix timestamp (in milliseconds), which corresponds to the timestamp of the first sample in each file. This timestamp is used to retrieve the timestamps of the remaining samples (please refer to Usage Notes for details). The waveform content of each file (after decompression) is described in Table 2.

**Table 2:** Description of the audio channels in the raw aggregated consumption files (<initial_timestamp>.w64).

Column	Description	Units
channel 1	Voltage	*Volt*
channel 2	Current	*Amp*

#### Pre-processed data

The pre-processed data files are available under the folder “mains/P_Q_V_I”. These are made available in two formats: 1) Sony Wave 64 (sample rate of 50 Hz), and 2) CSV (1 Hz).

The waveform content of the W64 files (after decompression) is described in Table 3. The columns of the CSV files are described in Table 4. In both cases, the file name indicates the timestamp of the first sample.

**Table 3:** Description of the audio channels in the 50Hz pre-processed aggregated consumption files (<initial_timestamp>.w64). VAR: Volt-Ampere Reactive.

Column	Description	Units
channel 1	Active Power	*Watt*
channel 2	Reactive Power	*VAR*
channel 3	Voltage RMS	*Volt*
channel 4	Current RMS	*Amp*

**Table 4:** Column descriptions in the 1Hz pre-processed aggregated consumption files (<initial_timestamp>.csv). VAR: Volt-Ampere Reactive.

Column	Description	Units
timestamp	Timestamp (YYYY-MM-DD HH:MM:SS) when the record was collected (UTC)	*datetime*
P	Active Power	*Watt*
Q	Reactive Power	*VAR*
V	Voltage RMS	*Volt*
I	Current RMS	*Amp*

### Individual appliance consumption measurements

The files with data for individual appliance consumption are available in the “appliances” folder. For each appliance there is a CSV file, named using the <id>_<name>.csv convention, where <id> refers to the unique identifier of the appliance, and <name> is the appliance name. The underlying fields of the individual appliance consumption files are described in Table 5.

**Table 5:** Column descriptions for the measurements files (<id>_<name>.csv).

Column	Description	Units
timestamp	Timestamp (YYYY-MM-DD HH:MM:SS) when the record was collected (UTC)	*datetime*
power	Appliance power consumption	*Watt*

### Labels

The files with the appliance transition labels are available in the “appliances/labels” folder. For each appliance there is a CSV file named using the <id>_<name>_labels.csv convention. The underlying fields are described in Table 6.

**Table 6:** Column descriptions for the labels files (<id>_<name>_labels.csv).

Column	Description	Units
timestamp	Timestamp (YYYY-MM-DD HH:MM:SS) of the appliance transition	*datetime*
source	Source of this label (S: System, H: Human)	*text*

## Technical Validation

### Aggregated consumption

In the course of the deployment, the aggregated consumption data collection system had to be rebooted four times due to issues with the USB communication. At the end of the deployment, there was a total of 2263 W64 files, divided across six consecutive periods. In order to reduce the number of files, the consecutive hour-long files were merged into W64 files. Before merging, each hour-long file was pre-processed to ensure that it had the expected number of samples, i.e., 12800 × 60 × 60 samples. The cleaning and the merging were done using the dsCleaner Python library^37^.

**Fig. 4:**
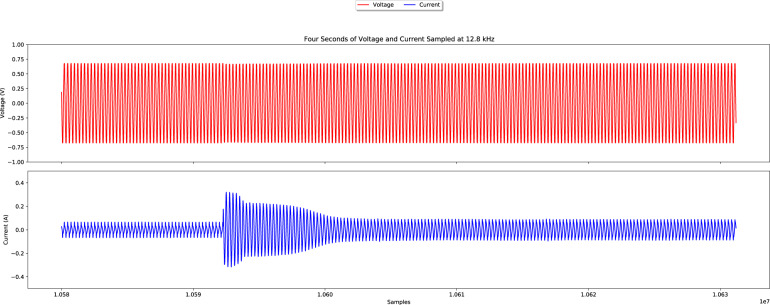
Four seconds of voltage and current sampled at 12.8 kHz.

As an illustration of the data contained in the raw current and voltage files, Fig. 4 depicts four seconds of the data in the file “mains/V_I/1477227096132.w64”. It is possible to observe an increase in the current signal corresponding to an appliance transition (the Freezer in this case.)

The aggregated voltage and current files were used to calculate the power metrics that comprise the pre-processed files. The calculations were originally performed at line frequency to obtain the 50 Hz files. Finally, the 1 Hz files were obtained by downsampling the 50 Hz files using the dsCleaner library. Technical details about the calculation of the power metrics are out of the scope of this data descriptor. But the interested reader can refer to^38^ (chapter 3).

**Fig. 5:**
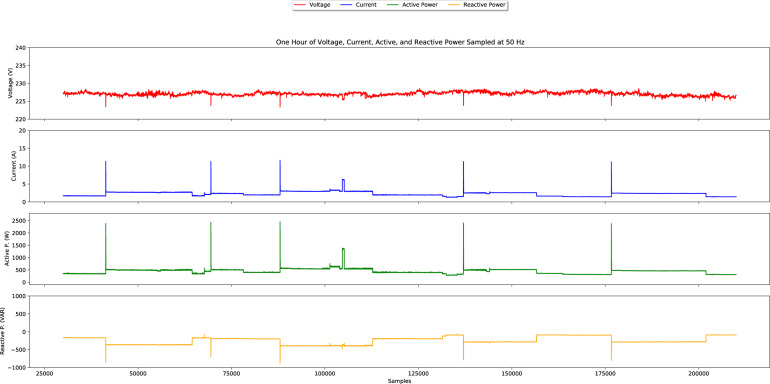
One hour of voltage, current, active and reactive power sampled at 50 Hz.

Figure 5 depicts one hour of aggregated consumption as it is stored in the raw processed files at 50 Hz (“1477227096132.w64”). As it can be observed, each file contains four channels: Voltage RMS, Current RMS, Active Power, and Reactive Power. It is also possible to see several appliance transitions, the first of which corresponds to the Freezer activation also observed in Fig. 4. Note also that in this case, the measurements are scaled to their original values using the calibration constants provided in the “calibration.txt” file.

### Individual appliance consumption

**Fig. 6:**
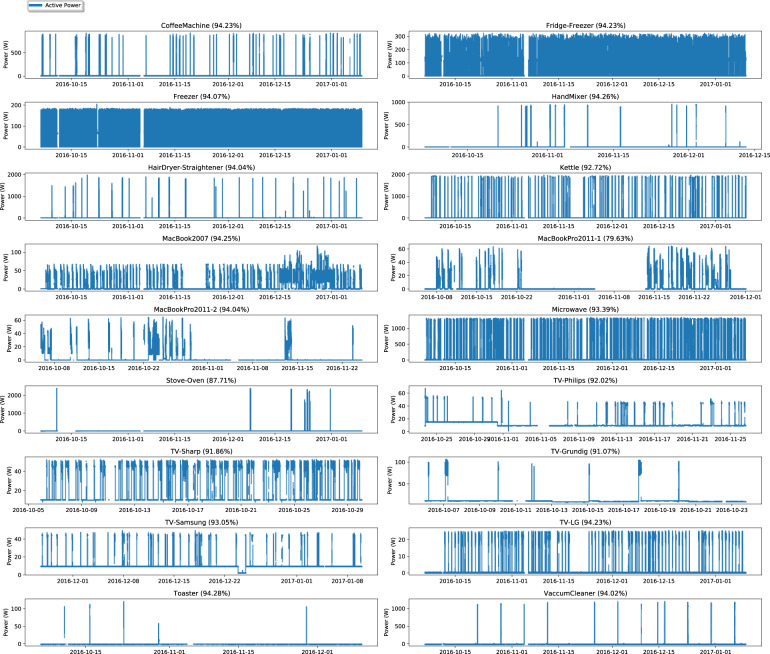
Graphical representation of the measurements obtained for each individual appliance for the entire duration of the deployment. The data is resampled to 0.5 Hz.

Throughout the deployment, between 2016-10-06 and 2017-01-09, there were 53,149.470 timestamped readings taken from the 18 appliances combined. Figure 6 depicts the measurements obtained from each plug for the entire duration of the deployment, resampled to 0.5 Hz, which was the actual rate of acquisition as mentioned in the methods section. As it can be observed, there are very few gaps in the data. In fact, on average, 92.3% of the expected samples were acquired (min: 79.6, max: 94.2, std: 3.4).

**Fig. 7:**
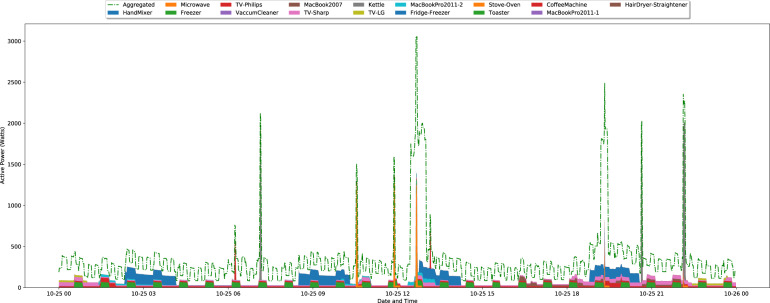
Graphical representation of 24 hours of aggregated and individual appliances consumption. The data is resampled to 1/60 Hz.

To further illustrate the collected ground-truth data, Fig. 7 depicts one day of aggregated consumption vs. consumption of the individual appliances resampled to 1/60 Hz. As it can be observed, there is a very good match between the aggregated and the ground truth. Still, even though the consumption for 18 individual appliances was collected, the amount of total energy explained is only about 38%. This happens due to the loads in the unmonitored divisions of the house. Such non-monitored appliances include a washing machine, water heater, iron, portable oven, and a second freezer. Table 7 summarizes the ratio between individual appliances and aggregated consumption for the entire duration of the dataset.

**Table 7:** Ratio between the consumption from the monitored appliances and the aggregated consumption for the entire duration of the dataset.

File	Period	Aggregated (kWh)	Appliances (kWh)	Ratio (%)
1475708700932.w64	2016-10-06 00:05 - 2016-10-22 13:45	161.7	60.0	37.2
1477227096132.w64	2016-10-23 23:51 - 2016-10-27 16:44	38.7	14.6	37.6
1477592018787.w64	2016-10-23 19:13 - 2016-11-11 15:27	129.0	41.0	31.8
1478884263362.w64	2016-11-11 17:11 - 2016-12-20 08:02	385.6	103.2	26.7
1482282276343.w64	2016-12-21 01:04 - 2016-12-31 16:11	132.2	38.1	28.8
1483205843836.w64	2016-12-31 17:37 - 2017-01-09 23:59	99.6	23.6	23.7

### Appliance labels

The labeling process results in a total of 12252 appliance labels from all appliances combined. Around 95% of these labels were obtained directly from the event detection algorithms, whereas the remaining 5% were added manually. The majority of the labels (70%) are from three appliances only, namely the Freezer (47%), microwave (14%), and fridge-freezer (7%). The number of labels per appliance is depicted in Table 8.

**Table 8:** Listing of the number of labels per appliance.

Appliance	Labels (S)	Labels (H)
Coffee Machine	312	2
Fridge-Freezer	1098	0
Freezer	5723	4
Hand Mixer	65	7
Hair Drier + Straightener	278	108
Kettle	463	4
MacBook 2007	824	110
MacBook Pro 2011 (1)	65	236
MacBook Pro 2011 (2)	20	50
Microwave	1701	55
Stove-Oven	398	2
TV Philips	91	1
TV Sharp	176	2
TV Grundig	18	0
TV Samsung	81	1
TV LG	231	26
Toaster	7	1
Vacuum Cleaner	79	13
	**11630**	**622**

**Fig. 8:**
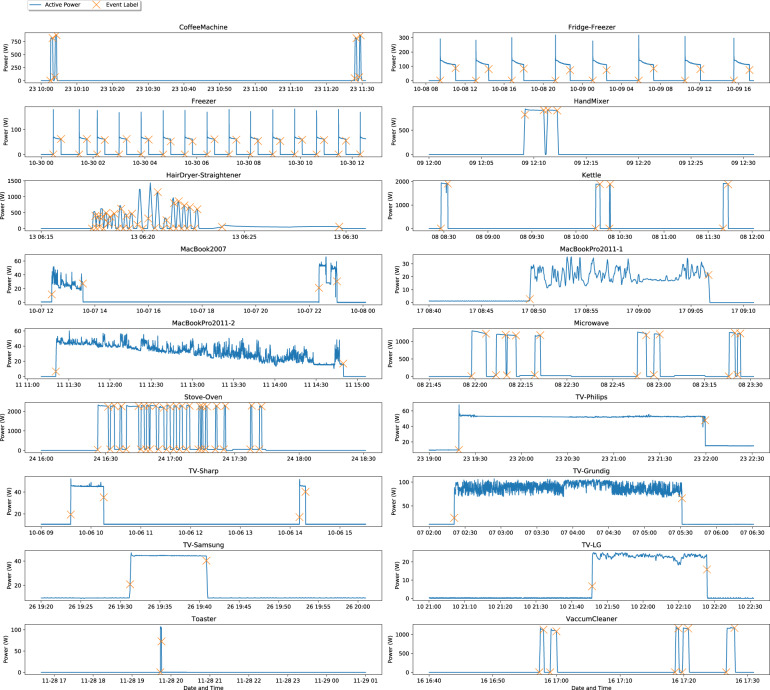
Individual appliances consumption supplemented with the respective transition labels.

Finally, to illustrate the ground-truth labels, Fig. 8 shows the consumption of each appliance supplemented with the respective labels. Note that for each label, it was necessary to find the respective power value on the consumption data since this is not available by default in the dataset.

## Usage Notes

### Decompressing files

The aggregated consumption data files are compressed using the WavPack Audio Compression format. Thus, before using the files, it is necessary to proceed with the decompression. The more straightforward way is using the wvunpack application directly from the command line. Alternatively, it is possible to use the WavePack decoders made available in different programming languages, including Java and C#.

### Reading files

The data are made available in W64 (after decompression), and CSV format, which are compatible with most software packages, including MATLAB, Python (e.g., dsCleaner and audiotools [see http://audiotools.sourceforge.net/, accessed 13/09/2021]), and Java (EMD-DF64^39^, and Java Sound API [see https://www.oracle.com/java/technologies/java-sound-api.html, accessed 13/09/2021]).

### Handling timestamps

#### Aggregated consumption

The aggregated consumption files stored in the W64 file format do not contain a timestamp. It is, therefore, necessary to calculate the timestamps, taking as input the timestamp of the first sample. This can be done individually for each sample using Eq. (1), which returns a Unix timestamp in milliseconds:

1 $$unix\_timestamp=1000\times \frac{current\_sample-1}{f}+initial\_unix\_timestamp$$

where *current_sample* is the position of the sample of interest, *initial_unix_timestamp* is the unix timestamp of the first sample, and *f* is the sampling rate of the waveform data. Alternatively, it is also possible to generate all the timestamps at once. For example, in Python this is possible using the pandas.date_range() command.

#### Appliances consumption and labels

Regarding the appliances consumption and labels, it is important to remark that the timestamps are represented in Universal Time Coordinated (UTC). Therefore, when converting the Unix timestamps to date and time formats, it is necessary to set the timezone to UTC to ensure that all the timestamps are always represented in the same timezone.

Furthermore, it is important to stress again the fact that the timestamps are not the same across all the appliances. Therefore, it is essential to align the timestamps before performing any operations on individual appliances. In Python, this can be easily achieved by resampling the data to 0.5 Hz and filling missing values using forward and backwards fill in sequence.

Finally, concerning the individual appliance labels, it is possible to convert the timestamps to an approximate sample in the aggregated data. This is done using Eq. (2):

2 $$position=\frac{actual\_unix\_timestamp-initial\_unix\_timestamp}{\frac{1}{f}\times 1000}$$

where *actual_unix_timestamp* is the Unix timestamp of the labelled transition to the mapped, *initial_unix_timestamp* is the timestamp in milliseconds of the first sample in the aggregated consumption, and *f* is the sampling rate of the aggregated consumption. Note, however, that since the individual appliance consumption is only available at 0.5 Hz, the obtained position can be delayed by up to two seconds.

## Data Availability

The code used to collect and store the aggregated consumption data is available at https://gitlab.com/alspereira/EMD-SF. This project used the EMD-DF library to create the audio files, which is available https://gitlab.com/alspereira/EMD-DF. The code runs using Java 8 or higher on a Windows machine. The code used to collect the individual appliance consumption is available at https://gitlab.com/mikemx55/Plugwise-2-M-ITI. The code runs using Python 3 on a Ubuntu machine. Finally, the Python 3 code to reproduce the examples presented in this paper is available on the dataset repository at https://osf.io/jcn2q/^36^.
